# Exercise Delays Brain Ageing Through Muscle‐Brain Crosstalk

**DOI:** 10.1111/cpr.70026

**Published:** 2025-03-24

**Authors:** Shirin Pourteymour, Rakesh Kumar Majhi, Frode A. Norheim, Christian A. Drevon

**Affiliations:** ^1^ Department of Nutrition, Institute of Basic Medical Sciences, Faculty of Medicine University of Oslo Oslo Norway; ^2^ Tissue Restoration Lab, Department of Biological Sciences and Bioengineering, Mehta Family Center for Engineering in Medicine Indian Institute of Technology Kanpur Kanpur India; ^3^ Center of Excellence in Cancer, Gangwal School of Medical Science and Technology Indian Institute of Technology Kanpur Kanpur India; ^4^ Vitas Ltd Oslo Norway

**Keywords:** brain, brain ageing, CNS myelination, exercise, healthy ageing, myokines and brain health

## Abstract

Ageing is often accompanied by cognitive decline and an increased risk of dementia. Exercise is a powerful tool for slowing brain ageing and enhancing cognitive function, as well as alleviating depression, improving sleep, and promoting overall well‐being. The connection between exercise and healthy brain ageing is particularly intriguing, with exercise‐induced pathways playing key roles. This review explores the link between exercise and brain health, focusing on how skeletal muscle influences the brain through muscle–brain crosstalk. We examine the interaction between the brain with well‐known myokines, including brain‐derived neurotrophic factor, macrophage colony‐stimulating factor, vascular endothelial growth factor and cathepsin B. Neuroinflammation accumulates in the ageing brain and leads to cognitive decline, impaired motor skills and increased susceptibility to neurodegenerative diseases. Finally, we examine the evidence on the effects of exercise on neuronal myelination in the central nervous system, a crucial factor in maintaining brain health throughout the lifespan.

## Graphical abstract

This graphical abstract illustrates the reciprocal crosstalk between the brain and peripheral organs, highlighting the profound impact of exercise on brain health through systemic interactions. It underscores how exercise influences the brain via signals from various organs and, conversely, how the brain modulates functions of peripheral organs. This integrative perspective sheds light on the bidirectional communication that underpins the interplay between physical activity, brain function and systemic health.

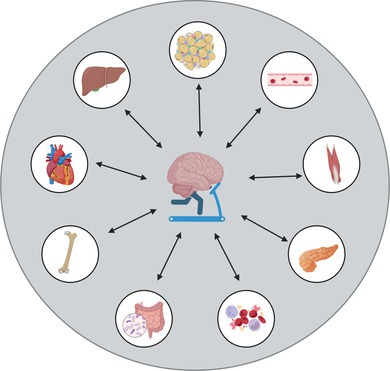

## 1 Introduction

### 1.1 Exercise and the Brain

Understanding the mechanisms underlying brain ageing can aid in the prevention or even the reversal of progressive cognitive decline, such as in dementia. Dementia encompasses a broad group of disorders characterised by a gradual decline in cognition, memory loss, language deficits, visuospatial impairment, reduced executive function and alterations in mood or behaviour [1, 2]. Approximately 47 million people globally are living with dementia‐related diseases, a figure projected to triple by 2050 [3]. Alzheimer's disease (ad), a prevalent form of dementia, affects approximately 6% of the population over 65 and becomes more common with age [4]. About 30% of ad cases can be attributed to modifiable risk factors, including hypertension, obesity, diabetes, and physical inactivity [3]. Dementia is associated with changes in the brain vasculature, size, morphology and signalling pathways [5]. Age‐related atrophy of the grey matter [6, 7, 8, 9], along with hippocampal shrinkage, is commonly observed and correlates with progressive memory loss [10, 11]. These changes may further contribute to a marked decline in learning capacity [12]. At the cellular level, synaptic contacts weaken, plasticity decreases [13], and hippocampal neurogenesis decreases [14, 15]. Although some degree of memory loss is a common consequence of ageing, it is not an inevitable outcome. The incidence of dementia increases with age, and cardiovascular diseases (CVD) enhance the risk of cognitive impairment [16, 17, 18, 19]. Thus, the prevention and treatment of CVD reduce the risk of dementia markedly [20]. This connection is demonstrated in mice with cardiac‐selective overexpression of adenylyl cyclase type 8 (TGAC8), which exhibit elevated heart rate and contractility, along with altered neuroautonomic surveillance. These TGAC8 mice demonstrated significantly enhanced locomotor activity, evidenced by a 43% increase in distance travelled, a 38% increase in average speed and a 45% reduction in freezing time. Moreover, in the hippocampus of these mice, key neurotransmitter receptors are upregulated, indicating higher mental activity. More specifically, the brain perceives the increased myocardial humoral and functional output as a ‘sustained exercise‐like’ scenario, prompting a response that activates central nervous system (CNS) output controlling locomotion. This response highlights how the heart–brain axis can play a significant role, with cardiovascular health being important for brain ageing [21].

There has been a significant shift in human lifestyle to a sedentary life over the past few centuries, contributing to the rise of lifestyle‐associated diseases [22]. Exercise is essential for maintaining both metabolic and mental health [23]. The numerous health benefits of exercise are widely acknowledged, including its positive effects on obesity, type 2 diabetes, cardiovascular diseases, osteoporosis, depression, dementia, sleep disturbances, non‐alcoholic fatty liver disease and various cancers [24, 25, 26, 27, 28]. Obesity, particularly in conjunction with social stress, impacts hippocampal structure and function, leading to reduced cognitive capacity partly due to diminished local pools of BDNF [29]. Exercise also reduces metabolic risk factors such as insulin resistance, blood lipid levels and chronic inflammation [30, 31, 32]. In contrast, 5 days of bed rest exert negative effects on muscle mass, insulin sensitivity, blood lipids and blood pressure [33]. Studies have shown that replacing sitting with standing during working hours or engaging in short bouts of light or moderate intensity walking between prolonged sitting may improve health outcomes, including increased plasma HDL levels and improved postprandial glucose and insulin levels [34, 35]. Exercise significantly influences human health, beginning in foetal development and continuing throughout the lifespan. Parental physical activity, both before and during pregnancy, influences the health of the mother and offspring [36, 37]. Maternal exercise during pregnancy is associated with benefits such as improved pregnancy outcomes, including reduced risk of macrosomia [38, 39], improved newborn neurobehavioural function [40] and cardiac autonomic health [41]. Furthermore, children of physically active mothers are more likely to adopt active lifestyles, lowering their risk of obesity and metabolic syndrome from infancy to adulthood [42]. Exercise during lactation also improves breastmilk composition, offering protection against obesity and inflammation [43] while supporting offspring brain development.

Large observational studies that track participants over time show that healthy adults who engage in regular exercise are less likely to develop dementia compared to inactive individuals [44, 45]. Exercise plays a crucial role in shaping our brain size, structure, and improves cognitive abilities [46]. A positive correlation between aerobic capacity and brain size has been reported [47]. Staying physically active throughout ageing promotes CNS function and reduces neuroinflammation as well as the risk of developing neurodegenerative diseases [48]. A systematic review indicates that resistance exercise induces significant functional alterations in the brain, especially in the frontal lobe, promoting enhanced executive functions [49]. Exercise is also associated with reduced white matter atrophy and smaller volumes of white matter lesions [49]. Additionally, exercise positively impacts grey matter volume and cognitive function in late adulthood [50, 51, 52, 53]. Age‐related decline in cortical regions appears particularly responsive to exercise [54, 55]. An atlas of exercise‐induced brain activation in mice reveals 255 brain regions activated by acute exercise, many of which were previously unlinked to exercise. Among these, 140 regions respond to both wheel and treadmill running, whereas 32 are unique to wheel running and 83 to treadmill running. Notably, forced treadmill running activates regions associated with stress, fear and pain [56].

**FIGURE 1:**
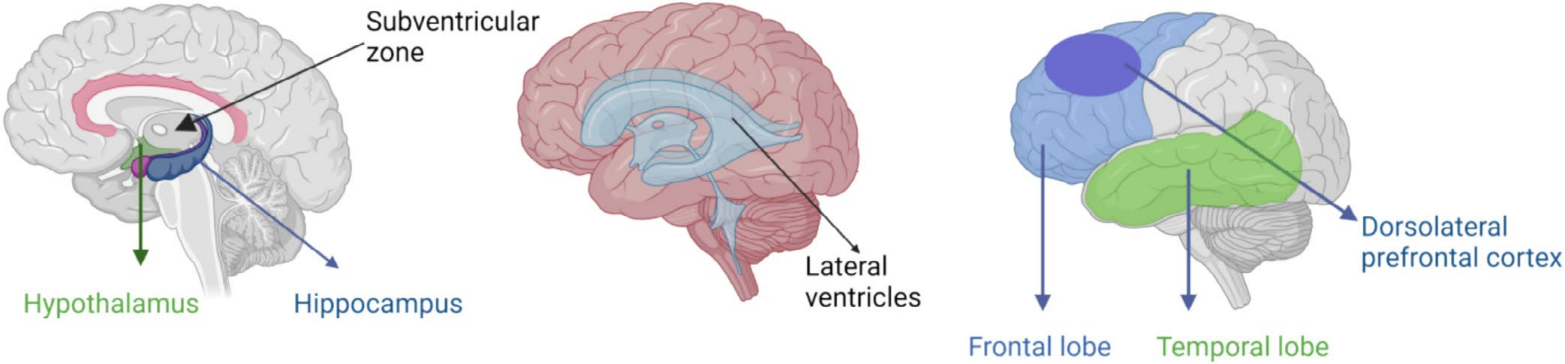
Brain regions associated with exercise. Exercise promotes growth in areas of the subventricles, hypothalamus and dorsolateral prefrontal cortex. The hypothalamus plays a crucial role in managing emotions, regulating body temperature, and controlling basic needs like eating and sleeping. The hippocampus has a major role in storing and retrieving memories, ‘sending’ memories to appropriate sections of the cerebrum for storage and retrieval.

Reduced blood–brain barrier (BBB) integrity in the human hippocampus is associated with early brain ageing and may be a contributing factor to cognitive impairment [57]. This may lead to hippocampal atrophy, which is also observed in ad [58, 59]. The hippocampus is known for its high degree of plasticity and neurogenesis, which provide an opportunity to enhance memory by improving hippocampal function [60]. For example, aerobic exercise has been shown to increase neurogenesis not only in the hippocampus but also in the hypothalamus and the subventricles [61, 62, 63, 64, 65, 66, 67]. Located along the lateral walls of the brain ventricles, the subventricular zone exhibits neurogenesis by producing new neurons in the adult brain [68]. A study of 115 individuals aged 50–70 years revealed that women who engaged in high levels of exercise had significantly larger volumes of the dorsolateral prefrontal cortex and temporal lobe compared to controls. Similarly, men who participated in high levels of exercise exhibited larger volumes in the temporal lobe [69]. The dorsolateral prefrontal cortex, which is positively influenced by exercise, plays a crucial role in cognitive functions like attention switching, working memory, rule maintenance, and inhibition of inappropriate responses [70] (Figure 1).

In old subjects, regular exercise significantly reduced brain tissue loss as compared to sedentary adults [71], and physically active old individuals have higher cognitive ability than sedentary old individuals of the same age [72, 73, 74, 75]. Physical activity includes any energy‐expending movement, whereas exercise is specifically structured and intentional, aimed at improving fitness. In this review, we use ‘exercise’ to denote purposeful physical activity.

## 2 Potential Mechanisms of Action

**FIGURE 2:**
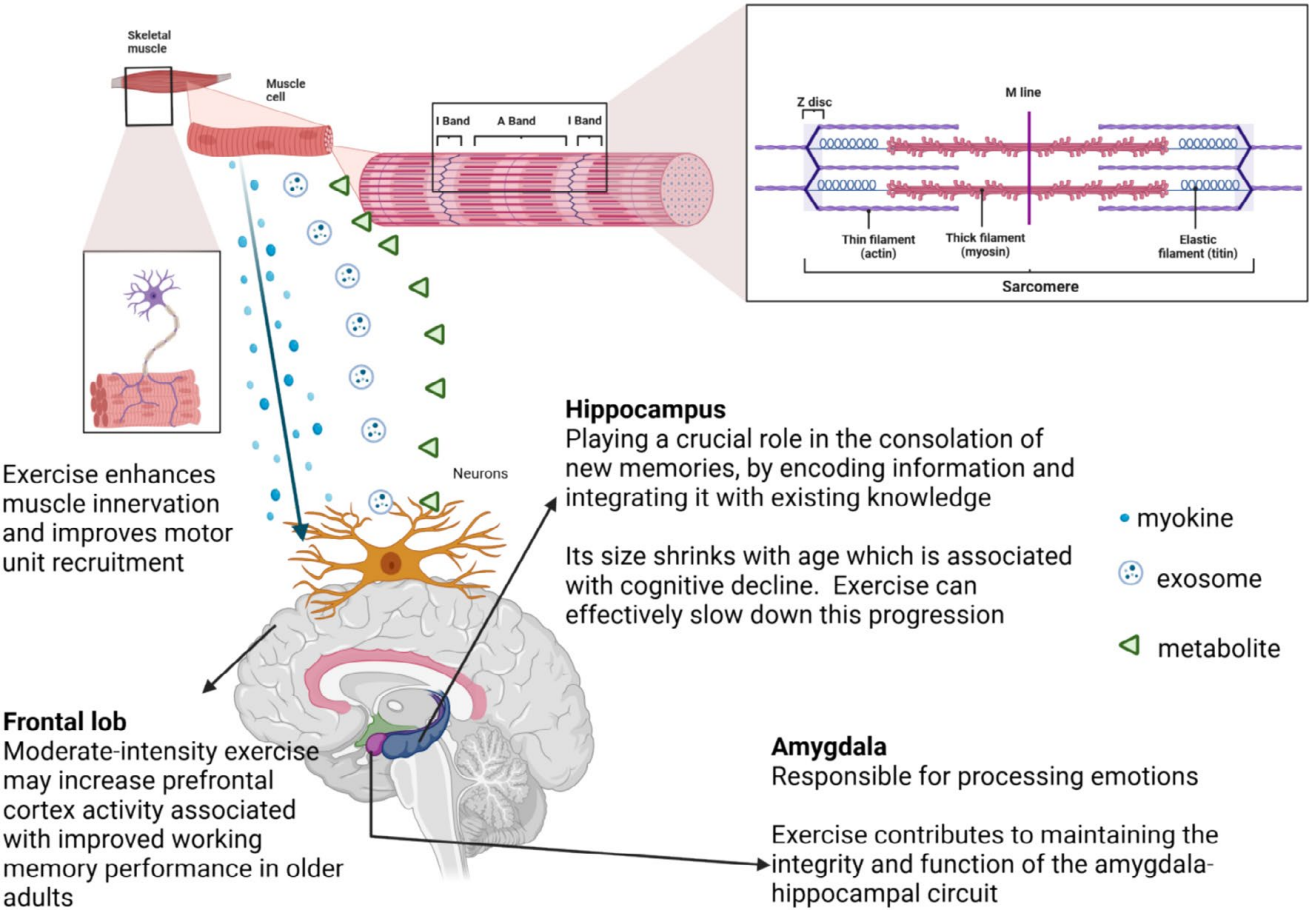
Skeletal muscle communicates with other organs via nerves and secretory proteins (myokines), metabolites, and extracellular vesicles (exosomes) released into the extracellular space and the circulation [79]. Exercise helps preserve the integrity and function of the amygdala‐hippocampal circuit [81]. Exercise‐induced improvement in hippocampal function may promote better memory and cognition [73]. Moderate‐intensity exercise may increase prefrontal cortex activity associated with improved working memory in older adults [82].

Skeletal muscle has been identified over the past decades as a hub for the production, secretion and release of myokines, which are defined as secretory proteins. Myokines may function as hormones with local effects (autocrine or paracrine) or affect distant cells and organs through endocrine effects [76]. Muscles may communicate with the brain via extracellular vesicles [77], myometabolites such as lactate [78], enzymes like cathepsin B and amylase [79], and indirectly via other organs like the liver releasing the ketone body beta‐hydroxybutyrate [80] (Figure 2).

All these pathways can modulate cerebral variables such as blood flow, metabolic rate, mitochondrial biogenesis, neurogenesis, protein folding, oxidative effects, inflammation, and cell senescence, thereby affecting mood, sleep, cognition, food intake, neurodegeneration, and development of brain‐related disorders [79].

We will examine selected myokines that may mediate the beneficial effects of exercise on brain health, particularly their roles in promoting neuronal myelination and regulating apolipoprotein E, a key factor in the accumulation of neuroinflammatory amyloid aggregates. Although irisin has been widely studied, we will not include it in this review due to ongoing debate about its existence as an exercise‐induced myokine with beneficial effects in humans [83, 84], as well as the availability of prior comprehensive reviews on this topic [85].

## 3 Myokines

The fact that plasma transferred from exercised to sedentary animals improves cognitive functions supports the presence of exercise‐induced circulatory factors [86, 87]. Exerkines are signalling molecules released in response to acute and/or chronic exercise, acting via endocrine, paracrine or autocrine pathways. These factors are produced by a variety of organs, tissues, and cells, including skeletal muscle (myokines), heart (cardiokines), liver (hepatokines), white adipose tissue (adipokines), brown adipose tissue (baptokines), and neurons (neurokines) [76]. Several exercise‐induced myokines have been described, including myostatin, interleukin‐6 (IL6). Myostatin was probably the first myokine to be described [88], whereas IL6 is the most extensively studied myokine in response to exercise or muscle contraction [89, 90].

### 3.1 Brain‐Derived Neurotrophic Factor (BDNF)

**FIGURE 3:**
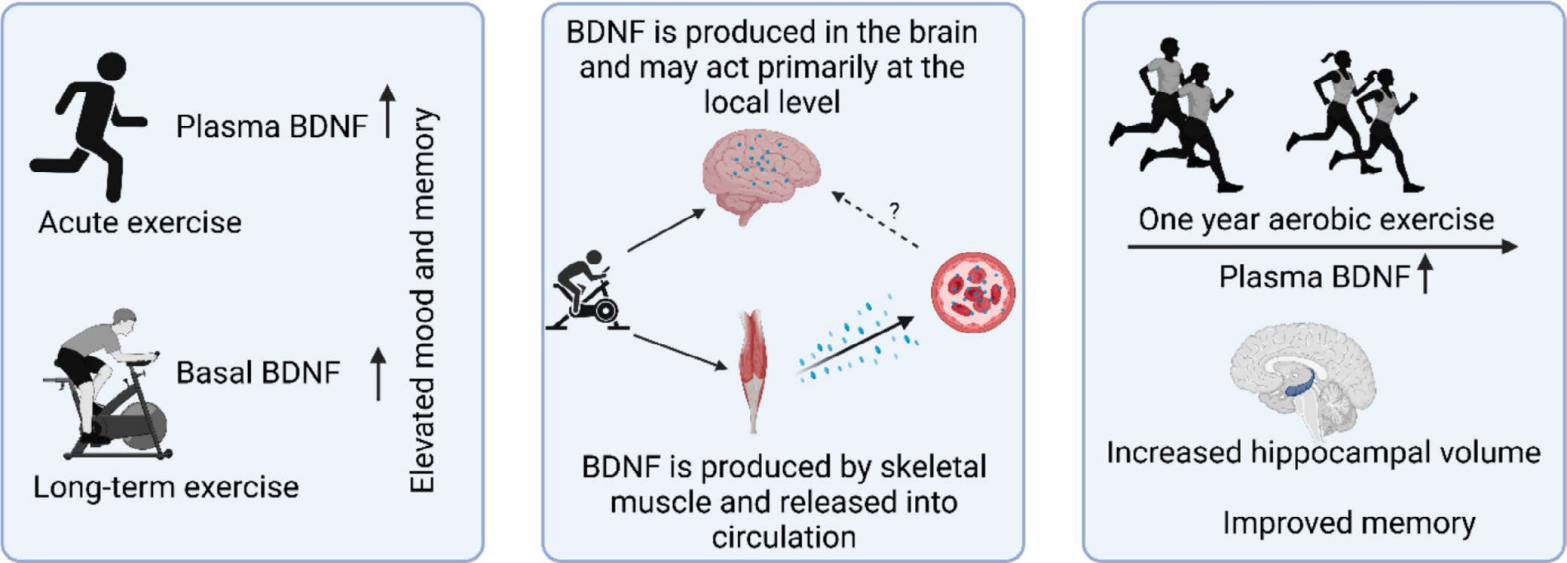
The role of BDNF in mediating cognitive effects of PA. Acute and long‐term exercise boost plasma BDNF levels, with long‐term exercise further augmenting basal BDNF levels, suggesting a potential for sustained effects. Exercise enhances BDNF levels in the brain and blood, but the mechanism by which blood BDNF may influence the brain is unclear. A year‐long exercise programme in older adults enhanced blood levels of BDNF, increased hippocampal volume, and improved memory performance [73].

BDNF (also called arbineurin) is an exerkine released by neurons, and partly also by skeletal muscle [91, 92] in response to exercise, especially aerobic activity. As a neurokine, BDNF highlights the systemic impact of exercise, benefiting both brain health and potentially other tissues through endocrine pathways [76]. It affects angiogenesis [93, 94], neuronal development, synaptic plasticity, growth and survival of neurons [95, 96]. BDNF is synthesised as a precursor protein (preproBDNF, ~34 kDa) and later cleaved to the 18 amino acid signal peptides to generate proBDNF (14 kDa), which is then transported to the Golgi apparatus for conversion into mature BDNF (BDNF; ~14 kDa) by proteases [97, 98] (Figure 3A). In contrast to most growth factors, certain processing products of proBDNF and BDNF pro‐peptide have biological functions often opposing those of the mature mBDNF [99, 100].

BDNF is one of the most highly expressed growth factors in the CNS [101]. It is expressed in several regions of the brain, particularly in the hypothalamus, cerebellum, amygdala, and the temporal lobe [102, 103, 104, 105]. It binds to the tropomyosin‐related kinase (TrkB) receptor, activates PI_3_K, MAPK, PLC‐γ, and GTP‐ases of the Rho family, modulating synaptic plasticity [103], and enhancing dendritic growth and synaptic plasticity, and perhaps episodic memory [106, 107, 108, 109], executive function, spatial memory, and learning [110, 111, 112].

Acute exercise significantly increases serum BDNF levels in concert with increasing exercise intensity [113, 114, 115, 116, 117]. Long‐term exercise also increases basal BDNF levels in serum [118, 119]. Randomised clinical trials have shown that aerobic exercise in schizophrenic patients enhances neurocognition and BDNF levels by nearly 15% in comparison to patients on regular psychiatric treatment [120]. Serum BDNF levels increase in Parkinson's disease patients by 34% after 8 weeks of moderate interval training [121]. Moreover, serum BDNF concentrations were increased in obese individuals after 30 sessions of aerobic exercise [122].

Several studies have linked high levels of exercise‐induced plasma BDNF to improved cognition [123, 124, 125, 126, 127]. Erickson et al. showed that a one‐year aerobic exercise programme enhanced hippocampal volumes, memory, and serum BDNF levels [73]. A cross‐sectional study assessed hippocampal volume, serum BDNF level and spatial memory in 142 participants (59–81 years of b), improved learning and memory in wild type and a Down syndrome mouse model [128]. Animal experiments indicated that BDNF may pass the BBB, and its blood levels may reflect the levels in the brain [129]. However, later studies have raised doubts about whether BDNF can pass the BBB or bind to its specific receptor on the BBB. Whether low, medium or high intensity exercise can affect the permeability of the BBB to BDNF remains to be ascertained. Notably, plasma concentration of mBDNF has been shown to increase during exercise [130, 131]. Mechanistic studies on its transport across the BBB are warranted to appreciate the beneficial effects of exercise‐induced BDNF on brain structure and function.

Myocyte BDNF production plays a crucial role in mitigating the severe effects of myocardial ischemia [132]. In addition, the functional BDNF/TrkB signalling axis is essential for proper myocardial function, as underscored by recent studies revealing its importance for maintaining cardiac health under stressful conditions [104, 133]. Yang et al. explored whether myocardial BDNF/TrkB signalling influences cardiac responses to pathophysiological stress [134]. They reported a significant reduction in BDNF levels in heart failure (HF) mouse models as well as humans with failing hearts. Interestingly, myocardial BDNF expression is increased in mice engaged in swimming exercise. Mice with a cardiac‐specific TrkB knockout exhibited a compromised adaptive response to swimming. These findings highlight the critical role of myocardial BDNF in modulating cellular responses to swimming, positioning it as a potential therapeutic target for enhancing cardiac function in HF [134]. Furthermore, BDNF levels decline with age, which may contribute to the deterioration of cardiac function observed in elderly individuals, further emphasising the importance of BDNF in age‐related cardiac pathophysiology [135].

### 3.2 Macrophage Colony‐Stimulating Factor 1

**FIGURE 4:**
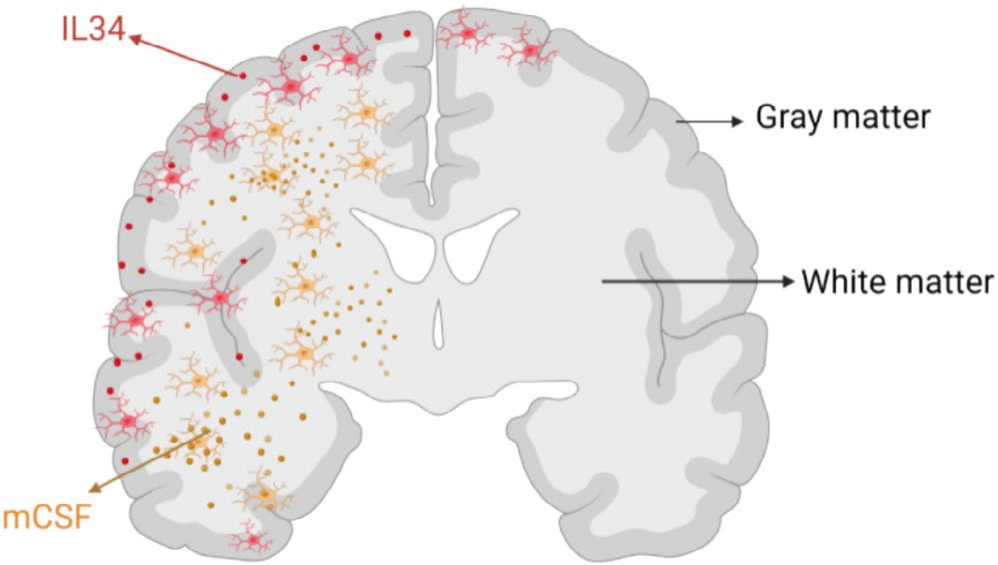
Muscle‐derived macrophage colony‐stimulating factor (mCSF) is produced during exercise and remains elevated post‐exercise. CSF1 affects a specific population of microglia in the brain in the white matter [138].

A series of transcripts regulated by acute and long‐term exercise has been described recently, which identified macrophage colony‐stimulating factor 1 (CSF1) as a secretory myokine upregulated by acute exercise as well as long‐term physical activity [136]. This was based on the measurement of CSF1 mRNA expression in skeletal muscle and its protein concentration in plasma before and after short‐ (hours) and long‐term (12 weeks) exercise in 26 sedentary men [137]. We have also simulated skeletal muscle contraction by electrical‐pulse stimulation in vitro and observed an increase in CSF1 mRNA in skeletal myocytes and CSF1 protein concentrations in the conditioned medium of cultured human skeletal myocytes [136]. These observations indicate that CSF1 is a secretory protein from skeletal muscle induced by exercise, and its production is increased in response to acute as well as long‐term exercise (Figure 4) [139].

Studies in humans and mice show that CSF1 exerts different effects on the brain via microglia density and microglia distribution in both white and grey matter [138, 140]. Microglia are resident myeloid cells in the CNS acting both as glial cells, preserving homeostasis with trophic support to neurons and other glial cells, and as immune cells carrying functions important in response to tissue damage [141]. The CSF1 receptor (CSF‐1R) belongs to tyrosine kinase receptors and is activated by the two homodimer glycoprotein ligands: CSF1 [142] and IL‐34 [143]. In contrast to CSF1, IL34 is not detectable in blood [144, 145], suggesting that its effects might be limited to its microenvironment. A specific CSF‐1R inhibitor given to mice led to a general depletion of microglia in the brain, whereas blocking the CSF1 and IL34 function with antibodies specifically reduced microglia density in the white and grey matter, respectively [138]. These data suggest that CSF1 may be of importance for linking some effects of exercise to the brain.

### 3.3 Vascular Endothelial Growth Factor (VEGF)

Skeletal muscle contributes to 60%–90% of peripheral vascular endothelial growth factor (VEGF) [146], and acute aerobic exercise transiently increases VEGF levels in muscle [147] and plasma [148, 149]. VEGF is a pro‐angiogenic factor promoting vascularization in several tissue types and may play a role in neurogenesis. Intravenous administration of a VEGF antagonist attenuates running‐induced hippocampal neurogenesis [150], whereas cerebroventricular administration of VEGF increases murine neurogenesis [151]. VEGF can cross the BBB, and most VEGF acting on the brain is probably derived from peripheral tissues [152]. By using a transgenic mouse deficient in skeletal muscle VEGF, it has been shown that VEGF deficiency reduces running‐induced hippocampal neurogenesis [146].

### 3.4 Interleukin 6 (IL6)

Contrary to CSF1, which maintains increased blood concentration also during long‐term exercise (12 weeks and 4 times per week of combined endurance and strength training), muscle‐secreted IL6 in plasma responds only to acute exercise and remains unchanged before and after 12 weeks of intervention [137]. Chronically elevated plasma IL6 in the resting state, on the other hand, seems to originate mostly from immune cells in adipose tissue [153]. The functions of IL6 include regulation of various biological processes associated with haematopoietic progenitor cells, adipose tissue, inflammation, hepatocytes, the placenta, the cardiovascular system, as well as the nervous and endocrine systems [154, 155, 156]. The expression of IL6 and IL6‐R has been observed in both central and peripheral nervous tissues, including glial and neuronal cells and sympathetic and sensory ganglia [157, 158, 159, 160, 161]. Long‐term excessive levels of IL6 may have negative effects on the homeostasis and chemistry of the nervous system. For instance, depressive patients and patients resistant to antidepressants have chronically elevated plasma IL6 concentrations in the CNS [162, 163]. Similarly, schizophrenic patients may have high plasma concentrations of IL6, and the severity of the disease is associated with the concentration of IL6 in the brain [164, 165]. Nevertheless, moderate and vigorous exercise may improve negative symptoms and brain function of schizophrenic patients [166].

### 3.5 Cathepsin B

Cathepsin B is another exercise‐responsive myokine [167] that may exert effects on the brain. Voluntary wheel running in cathepsin B knockout mice did not enhance hippocampal neurogenesis and spatial memory, opposite to the wild type, suggesting a role of cathepsin B in exercise‐associated cognitive improvements [168]. Cathepsin B and BDNF seem to be linked to long‐term exercise among men [169, 170], and cathepsin B may improve cognitive function by increasing peripheral levels of BDNF [168, 171]. Cathepsin B may execute its effects on the brain via autophagy, modification of neuroinflammation, synaptic plasticity, neurogenesis and metabolic regulation [172, 173].

### 3.6 Lactate

Although the brain only accounts for about 2% of the body weight, it receives ~20% of the total blood flow responsible for supplying oxygen, nutrients, hormones, and neurotransmitters, and removing carbon dioxide, toxins, and metabolic waste products [174]. Exercise‐enhanced angiogenesis and density of microvessels in the brain of ageing rats are associated with improved brain function [175]. High‐intensity exercise promotes anaerobic conditions in skeletal muscle, and glucose is shunted to glycolysis along with the release of lactate. Lactate can reach the brain and bind to a specific receptor named hydroxycarboxylic acid receptor 1 (HCAR1) on fibroblast‐like cells lining pial blood vessels (intracranial blood vessels on the surface of the brain), and may induce the expression of VEGFa [78] known for its role in angiogenesis [176], neurogenesis, synaptic transmission, and plasticity [177]. Thus, lactate may serve as a mediator of skeletal muscle–brain communication in angiogenesis, enhancing cerebral blood supply to support optimal cognitive performance and brain function.

## 4 Myelination

**FIGURE 5:**
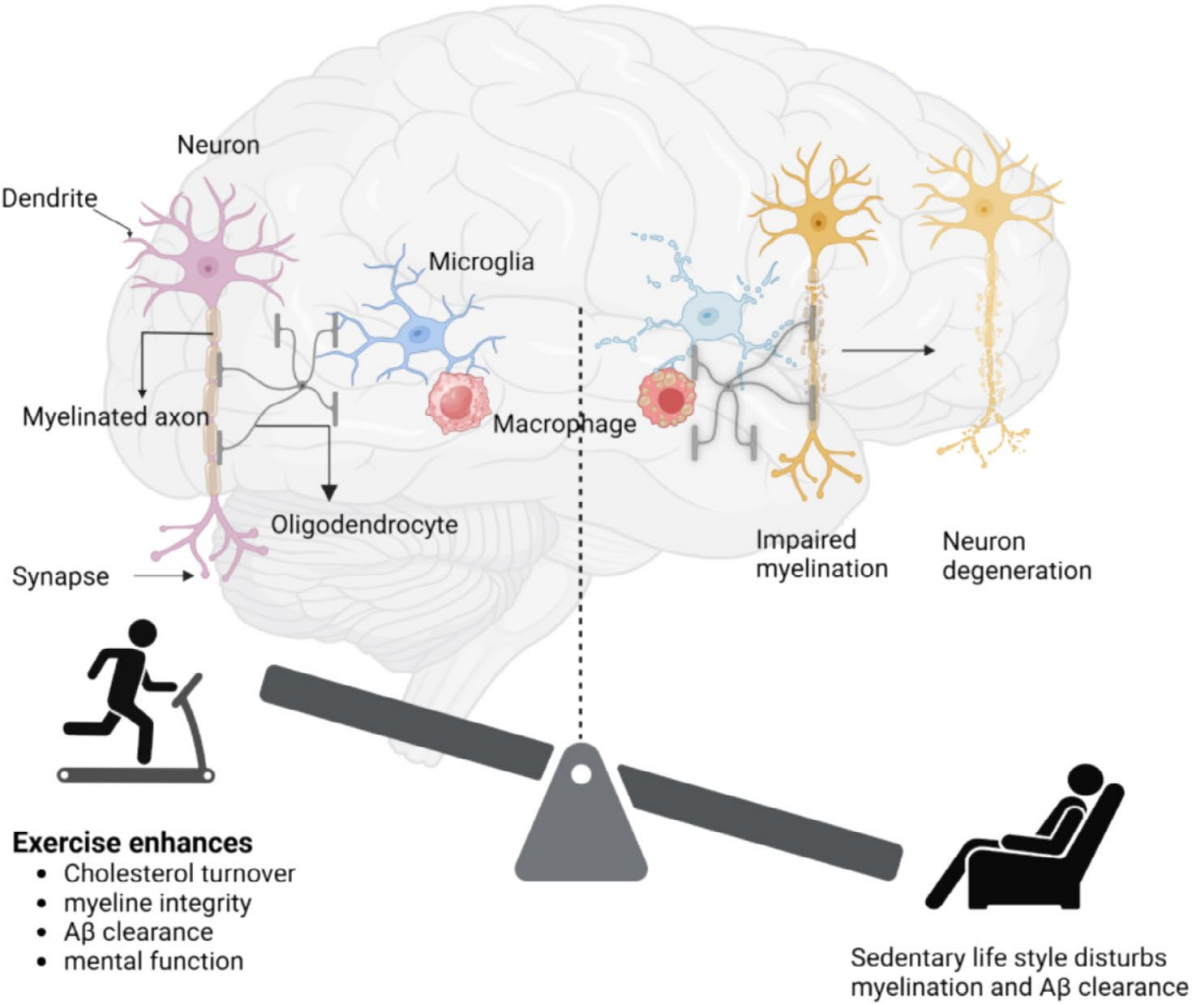
Physical activity may be beneficial for myelin formation and cholesterol homeostasis in the CNS through oligodendrocyte cells, which play an important role in both the synthesis and recycling of cholesterol required for the function of cell membranes and myelination.

Exercise also may affect white matter in the brain; therefore, we will discuss how myelination is linked to cholesterol metabolism and exercise (Figure 5). Myelination begins around birth in the peripheral nervous system, progressing to the spinal cord, and then in the brain throughout adulthood [178]. Myelination begins with the proliferation of oligodendrocyte precursor cells (OPCs) in the white matter. Then OPCs establish contact with axons and differentiate into myelinating oligodendrocytes.

Cholesterol increases myelin viscosity and stabilises myelin lipids and proteins, making it a critical and limiting factor for the development of myelin membranes in the CNS [179, 180]. BBB restricts the entry of peripheral cholesterol, meaning the primary source of brain cholesterol is local de novo synthesis in oligodendrocytes or astrocytes [181, 182]. Cholesterol in the CNS has a long half‐life, lasting about 1 year in mice and up to 5 years in humans, compared to just a few days in plasma [183, 184].

### 4.1 Exercise and Myelination in Rodents

Several studies have shown that exercise promotes myelination in rodents. Long‐term running exercise has been found to stimulate myelination in the motor cortex [185, 186], reverse toxin‐induced demyelination [187] and preserve myelinated fibres in brain white matter [188]. These findings are supported by a systematic meta‐analysis of 21 articles demonstrating the positive impact of exercise on myelin sheath regeneration in rodents [189].

A group of ‘depressive’ rats exhibited reduced length and volume of myelinated fibres, along with reduced volume and thickness of myelin sheaths [190]. However, the quality of myelin improved significantly following exercise compared to a control group [190]. Similarly, ad mice on a running programme for 4 months showed enhanced learning and spatial memory, as well as increased volumes of myelinated fibres in the CA1 region of the hippocampus [191].

### 4.2 Exercise and Myelination in Humans

A study including 88 healthy, untrained adults aged 60–78 years showed that engaging in exercise and avoiding sedentary behaviour enhanced myelin thickness in the brain [192]. Another study involving 10 sedentary subjects (74.5 ± 4.3 years of age) and 10 elite athletes (aged 72.2 ± 5.3 years, endurance training > 15 years) reported that life‐long exercise was associated with smaller lesions in brain white matter and better motor control and coordination [193]. Voss et al. examined the impact of a one‐year aerobic fitness intervention on the integrity of cerebral white matter and cognitive function in 70 adults aged 55–80 years [194]. The fitness program did not improve white matter integrity or cognitive function on a group level, but individuals with a higher score of aerobic fitness in the program experienced improved white matter integrity in certain brain regions and enhanced short‐term memory [194]. This study indicated that improved white matter integrity was not directly correlated with memory improvement but was associated with the extent of fitness gain [194]. Similar results were observed in a one‐year randomised controlled trial of aerobic exercise involving 36 patients with amnestic mild cognitive impairment [195]. A systematic review of 38 studies comparing cognitive and exercise training showed that cognitive training improved white matter microstructure, whereas exercise tended to enhance connectivity and larger structural outcomes concerning grey and white matter [196]. Some studies have reported a lack of correlation between exercise and myelination [197, 198]. However, it is important to note a limitation in these studies, as participants self‐reported their exercise levels through monthly telephone interviews [199].

### 4.3 Apolipoprotein E, Myelination, and Exercise

Apolipoprotein E (ApoE) is important in cholesterol homeostasis, with high expression in the liver and brain [200, 201, 202], and ApoE is associated with ad [203, 204]. In aged ApoE‐knockout mice, foamy glia cells (lipid‐loaded) accumulate in regions like the *thalamus*, *fimbria hippocampi*, and *hippocampus* associated with neurodegenerative and behavioural changes [205]. Long‐term aerobic exercise from midlife continued into old age mitigated neurovascular decline, reduced neuroinflammation, and enhanced synaptic plasticity and behavioural capabilities in mice [206]. The protective effects of exercise were linked to the preservation of astrocytic ApoE levels [206]. However, exercise had little effect on neurovascular decline or microglia activation in the absence of ApoE, suggesting that exercise may stabilise ApoE function [206].

ApoE2, ApoE3, and ApoE4 [207] represent common polymorphisms in the APOE gene; they play a significant role in lipid metabolism and CVD [208]. ApoE3 is the most prevalent variant in the general population, whereas ApoE4 constitutes the most significant genetic risk factor for ad [209]. These single amino acid polymorphisms modify the structure and function of ApoE, influencing its binding to lipids as well as receptors [210]. Prevalence varies by region, with APOE4 more common in certain populations, like Central Africa (40%), and less so in others, like South China (less than 10%). There is a gradient of APOE4 distribution in Europe and Asia, with higher prevalence in northern Europe and Asia (ca. 25%). APOE2 prevalence is higher in Africa and Oceania, with 9.9% and 11.1% penetrance, respectively. This variation may suggest selective advantages for specific alleles in different climates and populations [211, 212, 213].

Blanchard et al. [214] examined 32 post‐mortem brains (12 *ApoE3/3*, 12 *ApoE3/4* and 8 *ApoE4/4* carriers), 20 with ad, and the total group included 20 ApoE4 carriers. Using single‐nucleus transcriptional profiling, they identified affected pathways related to cholesterol synthesis in oligodendrocytes (ODC) in ApoE4 carriers. Analysis of the hippocampus and prefrontal cortex revealed cholesteryl ester accumulation in *ApoE4* carriers and reduced myelination, suggesting issues with cholesterol incorporation into myelin [214]. They also investigated the impact of ApoE4 on oligodendrocytes by creating human oligodendrocytes from induced pluripotent stem cells with engineered ApoE4 or ApoE3. They observed high cholesterol accumulation in ApoE4‐carrying oligodendrocytes, particularly around the endoplasmic reticulum (ER). This cholesterol accumulation induced ER stress and promoted nuclear translocation of the stress‐activated transcription factor ATF6 [214]. Promoting cholesterol transport with cyclodextrin reduced cellular cholesterol accumulation, potentially incorporating cholesterol into myelin [214]. These results are compatible with dysfunctional myelination in asymptomatic *ApoE4* carriers and reduced myelin levels in infants with ApoE4 [215, 216].

Amyloid‐beta (Aβ) is predominantly synthesised in neurons by proteolytic cleavage of amyloid precursor protein. The brain employs multiple pathways for removal of Aβ, including (a) cellular uptake and degradation; (b) enzymatic degradation; (c) clearance via the BBB; (d) clearance via interstitial fluid (ISF) bulk flow and (e) the glymphatic pathway. Cellular uptake of Aβ is facilitated by receptors like LDL receptor‐related protein 1 (LRP1), LDL receptor (LDLR), and heparan sulphate proteoglycan (HSPG) [217]. ApoE, primarily synthesised and lipidated by astrocytes, plays a crucial role in the clearance of Aβ. A subpool of ApoE‐containing lipoprotein particles interacts with soluble Aβ released from neurons into the brain ISF. Elimination of soluble Aβ from brain ISF takes place in an ApoE isoform‐dependent manner, where ApoE4 displays lower efficacy compared to ApoE2 or ApoE3 [218]. Abnormal myelination in the hippocampus may occur even before aggregation of amyloid and tau in ad mice [219]. It is possible that ApoE4 may compromise cholesterol efflux/metabolism, promoting impaired myelination, which may induce cholesterol accumulation in ODC, transforming them into foam cells.

**FIGURE 6:**
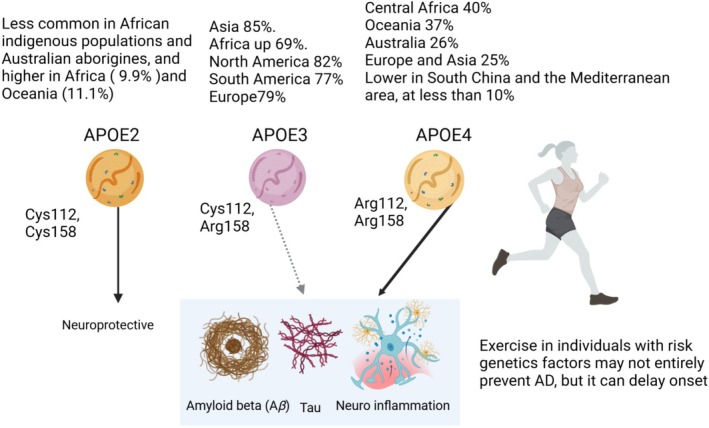
APOE alleles and their effects on CNS. APOE3 is the most common isoform, making up about 80% of alleles globally. APOE2 and APOE4 are less common, constituting around 5%–10% and 10%–15%, respectively.

In a murine model of multiple sclerosis, it was shown that exercise may improve BBB integrity and influence brain cholesterol homeostasis [220]. Scientists have reported effects of exercise on cholesterol flux in animals [221, 222]; thus, exercise may stimulate cholesterol efflux from brain cells, mitigating formation of foam cells. This may support optimal function of ODC, glia cells and phagocytes, enhancing cholesterol turnover, improving myelination, and clearing Aβ (Figure 6).

## 5 Effect of Exercise on the Blood–Brain Barrier

The concept of BBB dates back to the late 19th century, founded on observations that dyes and biologically active substances did not impact the brain or behaviour unless directly injected into the CNS [223, 224]. The BBB is dynamic throughout life; for instance, the transport of amino acids by the BBB varies significantly from neonates to adults [225]. A recent study on 20,000 subjects explored the permeability of the blood–cerebrospinal fluid barrier and the BBB, revealing significant sex differences in barrier integrity across all age groups indicated by the (Cerebrospinal fluid) CSF/serum albumin ratio, a key biomarker of barrier function [226]. These findings indicated that males generally exhibit lower barrier integrity. Moreover, CSF reabsorption slows with age, contributing to higher CSF/blood albumin ratios. The extent to which this age‐related increase in albumin ratios is due to BBB leakage or reduced CSF reabsorption remains unresolved [227].

Imaging, especially dynamic‐contrast‐enhanced magnetic resonance imaging (DCE‐MRI), is a preferred method for assessing BBB function in humans. Even during healthy ageing without pathological cognitive decline, BBB disruption is evident in the hippocampus, as well as in grey and white matter [228]. This disruption correlates with cognitive decline often associated with healthy ageing, particularly in delayed recall [229], highlighting a connection between BBB integrity and certain cognitive changes associated with ageing.

Animal studies consistently show beneficial effects of physical activity on BBB structure and function [230, 231]. Heart failure is known to compromise BBB integrity, significantly contributing to autonomic nervous system dysfunction by increasing caveolin‐1 expression, enhancing vesicle trafficking and weakening tight junctions, thereby elevating BBB permeability [232]. This BBB dysfunction disrupts neural regulation and exacerbates the systemic effects of HF, whereas exercising Wistar rats restores BBB integrity by normalising caveolin‐1 expression, reducing vesicle trafficking and strengthening tight junctions, all lowering BBB permeability [232].

Insulin resistance within the CNS is often associated with cognitive impairments like ad (ref). A study with CD‐1 male mice showed that acute exercise increased insulin transport across the BBB and improved insulin vascular binding in the brain for both sexes [233].

A study demonstrates that long‐term exercise enhances amyloid‐β clearance by improving BBB function in 5XFad mice, a transgenic model of AD that overexpresses amyloid‐β (Aβ). Exosomes derived from exercised 5XFAD mice promote proliferation and upregulate the mRNA expression of PDGFRβ, ZO‐1, and claudin‐5 in primary brain pericytes and endothelial cells in vitro. PDGFRβ is essential for pericyte survival and BBB maintenance, while ZO‐1 and claudin‐5 are key tight junction proteins that regulate endothelial barrier integrity and selective permeability. Notably, these exosomes exhibit significant alterations in miR‐532‐5p levels, and when administered to sedentary mice or transfected into primary brain cells, they replicate the BBB improvements observed in exercised mice. These findings suggest that exercise‐induced exosome signalling enhances BBB function by stabilising pericytes and reinforcing tight junction integrity, which may contribute to improved amyloid‐β clearance and neuroprotection in ad [234].

The effects of aerobic exercise on BBB integrity were also evaluated in a rat model of multiple sclerosis, demonstrating that exercise may improve markers of BBB integrity and reduce neuronal apoptosis [235].

A human intervention study explored the anti‐inflammatory effects of exercise and taurine supplementation on BBB integrity, inflammation markers, and cognitive function in 48 elderly women for 14 weeks. Participants were divided into groups: (a) combined exercise training; (b) taurine supplementation; (c) both exercise and taurine or (d) a control group with no intervention. Exercise alone (a), as well as in combination with taurine (b), reduced inflammation and preserved BBB integrity. Importantly, the group receiving both interventions (c) exhibited a significant improvement in cognitive function by scores on the Mini‐Mental State Examination [236].

A 12‐week moderate‐intensity aerobic exercise program in 56 methamphetamine‐dependent individuals (aged 18–45) significantly improved neurofilament light chain and neuron‐specific enolase blood levels compared to standard detoxification, indicating enhanced neurological recovery and blood–brain barrier integrity in the exercise group [237].

The reviewed articles consistently demonstrate that exercise improved the structure and function of the BBB, irrespective of the underlying causes of BBB disturbance, which include ageing, insulin resistance, neurological disorders, heart failure and methamphetamine abuse. It is reasonable to conclude that exercise may represent a good strategy for improving BBB integrity and function.

## 6 Summary and Future Direction

**FIGURE 7:**
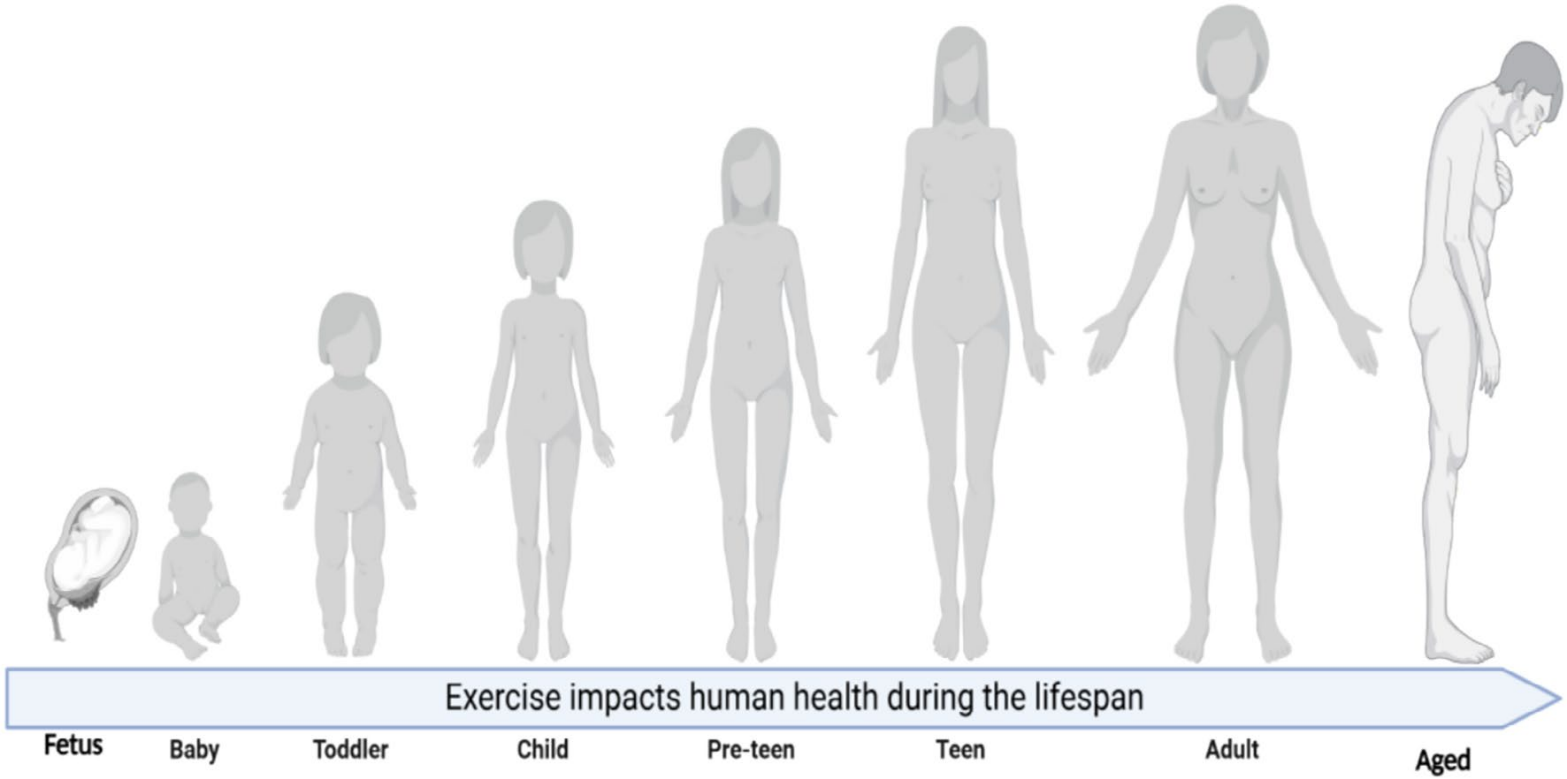
Exercise influences human health from foetal development to advanced age, highlighting the importance of physical activity throughout life. The benefits begin with maternal exercise, which positively impacts foetal development and extends into offspring health. Children born to physically active mothers tend to experience better health outcomes and are more likely to age healthily compared to those born to sedentary mothers.

Exercise confers significant benefits across all ages and sexes (Figure 7), improving brain‐related functions such as cognitive performance, mood regulation, sleep quality and mental health conditions like depression and dementia. These effects seem to be mediated by metabolic improvements including enhanced insulin sensitivity, reduced inflammation and cardiovascular health [238], as well as exerkines/myokines like BDNF (Figure 3), CSF1 [140, 143] and cathepsin B [171]. The influence of exercise on the heart‐brain axis is promising and merits deeper exploration [239].

The preventive and therapeutic potential of exercise enhances neuroplasticity and supports recovery, particularly in CNS disorders characterised by demyelination, like multiple sclerosis. The evidence underscores the importance of incorporating exercise into strategies designed to support ageing populations and manage neurodegenerative diseases.

Several questions still remain for future investigation. For instance, how do different types of physical activity affect specific CNS regions? What myokines and exerkines are produced in response to different forms of exercise, and how do these differ by exercise modality? Furthermore, how do male and female CNS and cardiovascular systems respond to exercise, and what are the differential effects of physical activity on the ageing CNS? Answers to these questions will advance our understanding of the mechanisms underlying exercise‐induced CNS benefits and refine targeted interventions for diverse populations.

## Author Contributions

Conceptualisation, manuscript preparation and revision: S.P., C.A.D. Manuscript review and revision: F.A.N., R.K.M. All authors have read and approved the final version of the manuscript.

## Conflicts of Interest

The authors declare no conflicts of interest.

## Data Availability

All data supporting this study are available in the published literature, as cited in the manuscript.
